# Retrospective Study on Antimicrobial Susceptibility of *Staphylococcus hyicus* Strains Isolated in Northern Italy Between 2022 and 2025

**DOI:** 10.3390/microorganisms14081733

**Published:** 2026-08-07

**Authors:** Benedetta Cordioli, Alice Prosperi, Camilla Torreggiani, Chiara Chiapponi, Carlo Rosignoli, Giorgia De Lorenzi, Simona Perulli, Giulia Dilio, Andrea Grassi, Antonio Marco Maisano, Andrea Luppi

**Affiliations:** Istituto Zooprofilattico Sperimentale della Lombardia e dell’Emilia-Romagna, Via Antonio Bianchi, 7/9, 25124 Brescia, Italy; alice.prosperi@izsler.it (A.P.); camilla.torreggiani@izsler.it (C.T.); chiara.chiapponi@izsler.it (C.C.); carlo.rosignoli@izsler.it (C.R.); giorgia.delorenzi@izsler.it (G.D.L.); simona.perulli@izsler.it (S.P.); giulia.dilio@izsler.it (G.D.); andrea.grassi@izsler.it (A.G.); antoniomarco.maisano@izsler.it (A.M.M.); andrea.luppi@izsler.it (A.L.)

**Keywords:** antimicrobial susceptibility, *Staphylococcus hyicus*, MIC, pig

## Abstract

Sudden outbreaks of exudative epidermitis (EE) are managed with antibiotics. This retrospective observational study evaluated the antimicrobial susceptibility of *Staphylococcus* (*S.*) *hyicus* strains isolated in Northern Italy between January 2022 and April 2025. Isolates were recovered from clinical cases submitted to IZSLER diagnostic laboratories. To ensure data independence, only one isolate per pig farm or strains from the same farm isolated at least 6 months apart were included in the final analysis. Strains resulted from bacteriological examinations on skin swabs (65) and synovial fluid (1) of pigs with EE. Antimicrobial susceptibility was assessed by determining the MIC of 16 antimicrobials in broth microdilution. MIC_50_ and MIC_90_ values were determined, and strains were classified as wild-type (WT) or non-WT according to EUCAST ECOFFs. Each antimicrobial showed different sub-populations with reduced susceptibility. MIC_50_ and MIC_90_ values were identical or differed by one dilution for most antimicrobials. The highest ratios of non-WT strains were penicillin (97%), tetracycline (91%), and clindamycin (90%). Conversely, non-WT strains ranged between 3 and 6% for oxacillin, cefazolin, ceftiofur, and rifampin.

## 1. Introduction

Exudative epidermitis (EE) is a globally distributed swine disease induced by *Staphylococcus* (*S.*) *hyicus* [1]. The bacterium is a Gram-positive coccus, and strains show variable virulence [1,2].

While *S. hyicus* is considered an endemic bacterium reported across major swine-producing regions—such as Europe, North America, and Brazil—clinical exudative epidermitis affects a substantial proportion of herds, with prevalence reaching approximately 30% of nursery pig farms in the United States [1].

Transmission is primarily by direct contact between animals of the same pen and between suckling piglets and the infected dam [3]. The development of EE involves the interplay of the virulence of the infecting strain, site of epidermal barrier disruption, and housing and management conditions (ex. ventilation). Moreover, the immune status of affected animals is crucial, as the disease is more frequent when maternal immunity is inadequate or absent. Consequently, outbreaks mainly affect piglets within the first eight weeks of life [1,3].

Following colonization of the stratum corneum, *S. hyicus* can breach the skin barrier, typically aided by predisposing factors such as physical trauma, solar dermatitis, or ectoparasitism [1]. Once the bacterium penetrates the deeper epidermal layers, the secretion of exfoliative toxins drives further tissue damage and bacterial proliferation. These toxins (most notably ExhA-D, ShetA and ShetB) represent the primary virulence factors of *S. hyicus* [1,2] and induce the separation of the strata granulosum and spinosum. By cleaving desmosomes, they create epidermal clefts that rapidly fill with inflammatory infiltrate and serous exudate, leading to the characteristic exfoliation of the outer epidermis and marked serous exudation [4]. Ultimately, the resulting loss of fluids and electrolytes causes severe dehydration, which can be fatal in affected piglets.

Although environmental improvements and autogenous vaccination are the main strategies to control persistent EE, sudden outbreaks are typically managed with injectable or in-feed/in-water antimicrobials. In some European countries (e.g., Denmark), surveillance programs are held to assess antimicrobial susceptibility of bacterial strains isolated from pigs. Moreover, reports from various countries document high levels of antimicrobial resistance of *S. hyicus* strains against different antimicrobial classes, with special attention to β-lactams such as oxacillin, which serves as a surrogate marker for methicillin resistance (MR) [5,6,7,8]. The occurrence of Methicillin-Resistant S. hyicus (MRSH) is a major concern as it confers cross-resistance to almost all β-lactam antibiotics, severely limiting therapeutic options for EE and presenting a potential zoonotic reservoir for resistance determinants.

In Europe, despite sharing a common legislative framework regarding veterinary antibiotics, critical variations in antimicrobial usage persist between nations. Indeed, notwithstanding significant recent reductions, Italy historically ranked among the highest consumers of antimicrobials for food-producing animals in Europe, preceded only by Spain and Cyprus according to ECDC reports from 2019 and 2021 [9]. However, data on the antimicrobial susceptibility of *S. hyicus* strains isolated in Italy remains unavailable. Thus, this study describes the in vitro antimicrobial susceptibility of *S. hyicus* isolated from clinically diseased pigs in Northern Italian pig farms from January 2022 to April 2025.

## 2. Materials and Methods

Isolates derived from 59 different pig farms were identified with alphabetical letters (A-Z, AA-AZ, BA-BS). Due to the retrospective nature of this study, sample size was determined by the availability of field isolates over the considered year period rather than a prospective power calculation. To avoid farm overclustering, one strain per farm or strains from the same farm isolated at least 6 months apart were included in the study. This 6-month interval was chosen based on the specific dynamics of Italian heavy pig production (rearing cycle of around 9 months). A total of 66 isolates were recovered, originating from 65 skin swabs and a joint swab. All samples were collected from diseased or dead pigs under 4 months of age showing clinical and gross lesions consistent with EE (generalized nonpruritic exudative dermatitis–epidermitis). These samples were derived from diagnostic submissions requested by farm veterinarians based on clinical suspicion of the disease. Skin swabs were collected from skin areas directly affected by clinical lesions of EE and submitted to the laboratory within 12 h of collection. When whole carcasses were delivered, animals had been deceased <4 h prior to submission, exhibiting acute clinical signs of EE. Table 1 summarizes the specimen type, isolation year and farm of origin for the strains included in the study.

Samples were plated on 5% sheep blood (BA) and Gassner (GA) agar plates, then incubated for 24 h at 37 ± 2 °C in aerobiosis. Plates showing delayed or insufficient growth were incubated up to 48 h to ensure maximum recovery of clinical strains and to confirm negative samples. Putative *S. hyicus* colonies (non-hemolytic with a white creamy appearance) were confirmed by matrix-assisted laser desorption/ionization–time-of-flight mass spectrometry (MALDI-TOF, Bruker Daltonics, Bremen, Germany). Isolates from pure cultures grown on Blood Agar (BA) for 24 h were tested according to the manufacturer’s instructions using the direct transfer method. An ethanol and formic acid protein extraction protocol was performed only when direct transfer failed to identify the strain. According to the manufacturer, identification scores were interpreted as follows: <1.7 indicated no reliable identification; 1.7–1.999 indicated probable genus-level identification; 2.0–2.299 = secure genus identification, probable species identification; and 2.3–3.0 = highly probable species identification. Isolates were tested in duplicate and scores ≥ 2.0 in both replicates were considered acceptable for species-level identification [10].

In vitro antimicrobial susceptibility was assessed by determination of the minimum inhibitory concentration (MIC). MICs of 16 antimicrobials were tested by broth microdilution in customized microtiter plates (ITISVE8, Thermo Fisher Scientific, Waltham, MA, USA) following the CLSI procedure [11,12]. Plates were incubated aerobically at 35 ± 2 °C for 18–24 h and read using the Vizion™ Digital MIC Viewing System (Thermo Fisher Scientific, Waltham, MA, USA). The MIC was defined as the lowest concentration of the antimicrobial that prevented visible growth. The concentrations of the antimicrobials tested in the commercial kit were amoxicillin/clavulanate (ratio 2:1) 0.25–16 μg/mL; ampicillin 0.03125–16 μg/mL; penicillin 0.03125–16 μg/mL; oxacillin 0.25–4 μg/mL; cefazolin 0.25–8 μg/mL; ceftiofur 0.25–8 μg/mL; clindamycin 0.5–2 μg/mL; enrofloxacin 0.25–4 μg/mL; erythromycin 0.03125–8 μg/mL; florfenicol 2–8 μg/mL; kanamycin 8–32 and 250–500 μg/mL; rifampin 0.625–2 μg/mL; tetracycline 0.25–16 μg/mL; tilmicosin 8–32 μg/mL; Sulfisoxazole 128–512 μg/mL; and trimethoprim–sulfamethoxazole (ratio 1:19) 0.125–8 μg/mL. *S. aureus* ATCC29213 served as a quality control strain and all MIC values fell within the acceptable limits defined by CLSI guidelines.

Strains were classified as wild-type (WT) or non-wild-type (non-WT) based on the Epidemiological Cut-Offs (ECOFFs) and tentative ECOFFs provided by the European Committee on Antimicrobial Susceptibility Testing [13] established for *S. hyicus*, and, when unavailable, those for *S. aureus* were used. In addition, isolates were further categorized as susceptible, intermediate or resistant based on clinical breakpoints (BPs). For data analysis, the overall resistance rate was calculated by combining both intermediate and resistant strains. Due to the absence of microorganism- and swine-specific clinical breakpoints for *S. hyicus*, classification relied on surrogate BPs derived from other staphylococcal species or hosts, which may not accurately reflect the pharmacokinetics/pharmacodynamics (PK/PD) of swine therapy. Specifically, dog-specific breakpoints (CLSI VET08) were applied to interpret susceptibility to ampicillin, cefazolin, enrofloxacin, clindamycin, and tetracycline [12]. CLSI human breakpoints were used for erythromycin, penicillin, oxacillin, rifampin, sulfisoxazole, and trimethoprim-sulfamethoxazole [11]. For ceftiofur and amoxicillin, interpretations were based on CLSI veterinary breakpoints for bovine mastitis and feline species, respectively. Finally, kanamycin MICs were evaluated according to the CASFM Vet 2019 standards [14].

MIC_50_ and MIC_90_ of each antimicrobial were evaluated considering the dilution able to inhibit the visible growth of 50% and 90% of the strains tested, respectively.

## 3. Results

The distribution of MICs of amoxicillin-clavulanate showed two sub-populations of strains, with a total of 57 strains at the concentrations <0.25 μg/mL (25 isolates) and 0.5 μg/mL (32 isolates). When oxacillin was tested, the strains exhibited a clear distribution across three sub-populations, with the vast majority clustering within the range of 0.5–2 μg/mL. Specifically, the MIC_50_ and MIC_90_ for this β-lactam were determined to be 0.5 μg/mL and 2 μg/mL, respectively. In comparison, cefazolin and ceftiofur showed similar MIC distributions, with most strains inhibited at 0.5 and 1 μg/mL, respectively. Considering penicillin and ampicillin, strains were distributed heterogeneously across the tested range, with 97% and 86% of the strains above the tentative ECOFFs, respectively. Similarly, trimethoprim–sulfamethoxazole MICs were distributed across the considered range, and 71% of isolates were classified as WT. The other sulphonamide tested (sulfisoxazole) showed a different MIC distribution: except for one isolate, strains were inhibited at the dilutions of <128 μg/mL (83%) and >512 μg/mL (15%). The distribution of clindamycin MIC was bimodal, with two antimicrobial dilutions separating the WT (7 strains) and non-WT (59 strains) sub-populations. Most of the isolates (57) were considered non-WT and were not inhibited at the highest concentration tested (2 μg/mL). Considering tetracycline, MIC distribution revealed three sub-populations, the largest placed at the dilutions of 16 and >16 μg/mL. Indeed, tetracycline was among the antibiotics with the highest ratio of non-WT strains (91%). Tilmicosin and erythromycin showed 82–84% of non-WT isolates, both with two sub-populations, divided by one and three dilutions, respectively. Moreover, kanamycin exhibited a clear bimodal distribution of MICs, with most strains placed at the concentrations of <8 μg/mL (40 isolates) and >32 μg/mL (17 isolates). When rifampin was tested, 62 out of 66 strains were inhibited at the lowest concentration of the range. Enrofloxacin inhibited 61/66 isolates at the lowest concentration tested (<0.25 μg/mL). The remaining strains were distributed across higher dilutions, with only 8% classified as non-WT. Florfenicol divided the isolates in two sub-populations: 19 isolates were inhibited at the lowest concentration tested (2 μg/mL), whereas the others grouped at the dilutions between 4 and >8 μg/mL.

For most of the antimicrobials tested, MIC_50_ and MIC_90_ values were either identical or differed by one dilution. Conversely, the difference between MIC_50_ and MIC_90_ of oxacillin and florfenicol was two serial dilutions, and the difference was even greater for trimethoprim–sulfamethoxazole, kanamycin and ampicillin.

Comprehensive results of antimicrobial susceptibility testing are summarized in Table 2, whereas Table 3 summarizes the annual proportions of non-WT strains for each tested antimicrobial with available ECOFFs and BPs.

## 4. Discussion

The growing challenge of antimicrobial resistance demands a rational use of antimicrobials in livestock. Consequently, when treatment is required, antimicrobial susceptibility testing is crucial to ensure that animals are treated with the effective active principle, thereby limiting the risk of resistance. While outbreaks of EE are not very common in Italy, antibiotics remain the primary option to control sudden outbreaks on farms, despite the lack of available data on the antimicrobial susceptibility of local *S. hyicus* strains.

Several limitations of this study should be noted. First, the analyzed isolates were considered clinical EE strains based on the anamnesis and the clinical–anatomopathological picture, whereas toxin production was not considered. Epidemiological studies demonstrate that toxigenic *S. hyicus* isolates are significantly more common in EE-affected pigs than in healthy controls [15,16]. Nevertheless, a recent survey conducted on Spanish strains revealed the presence of toxin-encoding genes in approximately 50% of the isolates regardless of the sample type or the clinical signs [8]. These observations suggest that while toxigenicity remains a key driver, clinical disease in the field is likely influenced by host susceptibility, environmental co-factors, and potentially uncharacterized virulence mechanisms. Furthermore, no significant correlation has been observed between toxin gene carriage and antimicrobial resistance profiles [17]. 

The relatively small sample size and the single-isolate-per-farm sampling strategy precluded advanced statistical modeling; thus, comparisons with international literature and the evaluation of temporal susceptibility trends were restricted to descriptive statistics. Furthermore, variations in study periods and swine target populations across published reports may have influenced these comparative findings. Another constraint lies in the current lack of *S. hyicus*-specific ECOFFs and swine-specific breakpoints (BPs) for several tested antimicrobials. Consequently, MIC values for oxacillin, cefazolin, ceftiofur, clindamycin, kanamycin, and rifampin were interpreted using *S. aureus* surrogates [13]. Overall, despite these limitations, Italian *S. hyicus* strains exhibited a broad spectrum of reduced susceptibility to the antimicrobials evaluated. Additionally, in this study only phenotypic resistance was characterized, leaving the underlying genetic mechanisms unexplored.

Among the tested β-lactams, ampicillin and penicillin showed the highest ratio of non-WT strains. Particularly, the prevalence of strains above the tentative ECOFF of ampicillin in our survey (86%) is considerably higher when compared to studies conducted in Brazil (28.15%) and Spain (30.3%) [7,8]. Regarding penicillin, the MIC_50_ in our study is a step higher than the MIC_50_ that resulted when Danish *S. hyicus* strains from 2012 to 2015 were tested, and the ratio of non-WT strains is higher than Spanish isolates [5,8].

Current Italian guidelines for swine antimicrobial treatment were originally drafted by the Emilia-Romagna region and subsequently adopted by the Italian Ministry of Health to support antimicrobial stewardship and disease management. Although penicillin, both in its injectable and oral formulation, is considered the first-choice antibiotic, its practical application in Italy remains limited [18]. Challenges include the lack of long-lasting formulas, the burden of frequent injections, and the unavailability of oral penicillin on the Italian market. Therefore, the observed reduced susceptibility may be associated with cross-resistance following exposure to ampicillin and amoxicillin, which are suggested as second-choice antibiotics for the treatment of EE [18]. Unfortunately, detailed records of previous antimicrobial treatments administered to the specific animals submitted or sampled for diagnostic investigation are often unavailable. Lacking farm-level treatment data, it remains speculative whether the proportion of non-WT strains for penicillin and ampicillin is associated with previous antibiotic exposure, including treatments directed at other conditions, such as streptococcosis.

The other β-lactams included in our study recorded the lowest ratios of non-WT strains, together with rifampin (3–6%). Notably, only two isolates exceeded the ECOFF of oxacillin, indicating a potentially low risk of methicillin-resistant (MR) *S. hyicus* among the tested isolates. However, the presence of sub-populations with reduced susceptibility warrants continued monitoring. Indeed, characterizing these isolates for the presence of *mecA* or novel *mecA* homologue genes is essential to map the genetic determinants driving these phenotypes. When classified according to veterinary clinical breakpoints, these strains would be considered resistant not only to oxacillin but to all β-lactams [12]. In addition, MR *Staphylococcus* strains often show poor clinical response to β-lactam therapy, and clinical data documenting clinical efficacy for those agents are currently lacking [19].

Our findings revealed a discrepancy in resistance profiles within the β-lactam class, characterized by high resistance rates to penicillin and ampicillin contrasting with higher susceptibility to amoxicillin-clavulanic acid. This specific phenotype is highly consistent with β-lactamase production mediated by the *blaZ* gene, an enzymatic resistance mechanism extensively documented throughout the genus *Staphylococcus* [20]. This enzymatic mechanism is further supported by the high susceptibility rates maintained against oxacillin, cefazolin, and ceftiofur. Because classical staphylococcal β-lactamases do not hydrolyze these molecules, the preservation of their in vitro activity strongly reinforces that resistance in our isolates is driven by enzymatic degradation rather than structural modifications, such as the production of a modified penicillin-binding protein (PBP2a) encoded by the *mecA* gene [21].

Resistance to ampicillin and penicillin remained close to 100% across the four-year period, confirming the ubiquity of beta-lactamase production within this species. Interestingly, amoxicillin + clavulanic acid exhibited a systematic misalignment between clinical and epidemiological interpretations: clinical resistance (R) remained high, ranging from 56% to 78%, whereas the non-wild-type (non-WT) sub-population never exceeded 24% (dropping to 0% in 2024). This divergence may derive from the classification of microbiologically WT strains, likely those falling into the “intermediate” category, as resistant applied in this study. Conversely, cephalosporins (cefazolin and ceftiofur) maintained high in vitro susceptibility, with resistance rates consistently below 12%, representing viable β-lactam alternatives. The consistently low prevalence of oxacillin resistance (≤6%), already described in other studies, indicates that methicillin-resistant *S. hyicus* (MRSH) remains rare in the sampled population, confirming that the therapeutic challenges observed are primarily driven by β-lactamases and mechanisms other than *mecA* acquisition [5].

Clindamycin was tested as representative of lincosamides, and our data (90% non-WT strains) is only slightly different from studies conducted on Spanish and Brazilian isolates. Notably, the latter study showed 99.03% resistance, despite using a higher cut-off value to classify the isolates [7,8].

Results of this study, considering tetracycline and macrolides, showed higher MICs than data available in the literature. Indeed, Danish isolates (2012–2015) revealed 44.4% of strains resistant to tetracycline, and more recent studies testing oxytetracycline and chlortetracycline showed resistant and non-WT ratio varying from 67 to 73% and from 28 to 29%, respectively [6,7,8]. Moreover, regional differences concerning erythromycin and tilmicosin can be detected by comparing our study with others that used the same cut-off value (Denmark and Spain). Both macrolides yielded a higher proportion of non-WT strains in our survey than in the Spanish (tilmicosin, 59%) and Danish (erythromycin, 33.3%) studies [6,8]. In a recent South Korean study applying a different threshold, interpreting tilmicosin MIC values using the ECOFF identified only 35% of strains as WT, a proportion that remains higher than what was observed in our study [22].

The high rate of concomitant resistance to erythromycin, tilmicosin and clindamycin observed in the tested strains is highly suggestive of the macrolide-lincosamide-streptogramin B (MLSB) phenotype. This mechanism is typically mediated by *erm* genes and determines modification to the antibiotic binding site. Also, it is the most well-described cross-resistance pathway across these three antimicrobial classes in Gram-positive cocci. Although the lack of molecular characterization to confirm the specific genetic determinants (*ermA*, *ermB* or *ermC*) represents a limitation of this study, the widespread prevalence of this phenotypic pattern is well-documented globally [23]. Notably, this resistance profile has been reported even in livestock populations with no prior history of exposure to these specific antimicrobial classes [24].

High resistance rates (>75–80%) were observed for clindamycin, erythromycin, and tetracycline throughout the years considered in the study. These findings may be associated with historical therapeutic pressure determining resistance traits for these classes within pig herds, rendering them unsuitable for empirical treatment of EE.

Overall, the prevalence of non-WT strains for enrofloxacin and florfenicol in this study is lower compared to other reports, in which the ratio of non-WT isolates ranges between 40 and 65% and between 49 and 74%, respectively [7,8,22]. Nevertheless, the detection of less-susceptible sub-populations underscores the need for continuous surveillance. Although the high susceptibility observed against these antimicrobials is a favorable finding, the presence of a sub-population with reduced susceptibility could reflect an intermediate stage of selective pressure, indicating an evolving resistance pattern.

A favorable downward trend was observed for fluoroquinolones, with enrofloxacin resistance dropping from 19% in 2022 to 0% in 2025. This sharp decline likely reflects the positive impact of restricted usage policies and strict veterinary regulations surrounding Critically Important Antimicrobials (CIAs).

In contrast, trimethoprim–sulfamethoxazole registered a higher prevalence of non-WT strains than other studies in the literature [5,7,8]. Notably, the heterogeneous distribution of MIC values for this antimicrobial likely suggests a variable selective pressure operating at farm level, probably reflecting differences in antimicrobial usage patterns among the sampled facilities. While most of these strains placed below the tentative ECOFF, this finding suggests a potential shift towards more resistant strains in the future, also because of the detection of enzymes with altered trimethoprim affinity detected in Staphylococci of animal origin [25]. However, a slight contraction in resistance was noted for trimethoprim/sulfamethoxazole (from 31% to 12%), although a methodological mismatch persisted here as well, with non-WT proportions systematically outnumbering clinical R rates (e.g., 50% vs. 31% in 2022).

Kanamicin was tested as a representative of the aminoglycosides. The clear bimodal distribution of the MICs suggests the presence of acquired resistance mechanisms within a sub-population of the tested strains (40% non-WT strains). In staphylococci isolated from livestock, aminoglycoside resistance is generally mediated by genes encoding aminoglycoside-modifying enzymes (AMEs), which enzymatically inactivate the antibiotic [25]. Among these, bifunctional enzymes or specific adenylyltransferases and phosphotransferases are frequently involved in resistance to kanamycin and other aminoglycosides in animal-associated staphylococcal species [25].

Our finding aligns with variable levels of susceptibility to aminoglycosides reported in international literature. For instance, resistance to gentamicin varies considerably across studies, ranging from 8% in Brazil (2012) to 20% non-WT strains in Spain [7,8]. Interestingly, Danish data (2012–2015) revealed resistance rates ranging from 0% for gentamicin to 33% for streptomycin, suggesting that reduced susceptibility among *S. hyicus* strains may be more pronounced for specific aminoglycosides [5].

Considering results as a whole, with the exception of amoxicillin-clavulanic acid and trimethoprim-sulfamethoxazole, the resistance proportions interpreted using CLSI clinical breakpoints (BPs) were largely comparable to the non-wild-type (non-WT) proportions. For trimethoprim-sulfamethoxazole, threshold discrepancies led to a lower proportion of resistant strains, as the clinical BP is three dilutions higher than the ECOFF. Conversely, for amoxicillin-clavulanic acid, 32 strains fell into the intermediate category, resulting in a 62% resistance rate.

Geographic heterogeneity in antimicrobial susceptibility profiles of *S. hyicus* strains is well established within the literature. Beyond divergences in nation guidelines and national surveillance programs, several drivers may account for these regional variations. Specifically, we speculate that indirect selective pressure plays a major role; treatments targeted at concurrent endemic swine pathogens (e.g., streptococcus) could determine co-selection of resistant *S. hyicus* strains. For instance, the extensive use of beta-lactams to manage streptococcosis within herds could collateralize the co-selection.

Furthermore, high herd density within major swine-producing regions can facilitate both the rapid horizontal transmission of resistant clones between facilities and the horizontal transfer of mobile genetic elements carrying resistance determinants. Finally, differences in regional biosecurity and hygiene protocols—including the varying use of specific disinfectants or topicals—may further shape the distinct antimicrobial resistance landscapes observed across different countries by driving co-resistance mechanisms.

Our results strongly support the need for antimicrobial susceptibility testing of the isolates responsible for clinical EE to ensure rational therapeutic protocols. If empirical therapy cannot be avoided, systemic administration via injection is recommended, and trimethoprim/sulfamethoxazole is a suitable choice [1]. Depending on in vitro susceptibility testing, ceftiofur and enrofloxacin may also be used. Antimicrobial resistance represents one of the most critical threats to both human and veterinary medicine worldwide. The complex and interconnected nature of this emerging challenge mandates a comprehensive One Health approach, emphasizing that human, animal, and environmental health are intrinsically linked. Within this framework, antibiotic susceptibility surveillance in livestock and food-producing animals is essential for monitoring resistance trends and preventing zoonotic transmission.

## 5. Conclusions

In conclusion, Italian *S. hyicus* strains revealed reduced susceptibility to a wide range of antimicrobials. Oxacillin, cefazolin, ceftiofur and rifampin showed the highest prevalence of WT strains. On the contrary, penicillin, tetracycline and clindamycin showed high levels of non-WT strains, possibly due to selective pressure following antibiotic exposure for the treatment of other diseases. In the future it would be critical to continue monitoring the susceptibility of clinical *S. hyicus* strains against antimicrobials, particularly CIAs and ß-lactams. Moreover, the correlation of the phenotypic resistance with its underlying genetic determinants will provide insightful information to fully understand the molecular epidemiology of the isolates. Specifically, our phenotypic findings highlight the need to monitor *blaZ* and *mecA* genes when assessing ß-lactam susceptibility, alongside *erm* genes in strains exhibiting the MLSB phenotype.

## Figures and Tables

**Table 1:** Description of the specimen, year and farm of isolation for the *S. hyicus* strains included in the study.

Specimen	Year	Nr. of Isolates	Farm
Joint swab	2022	1	A
Skin swab	2022	15	B-R
	2023	9	S-AD
	2024	24	L, V, T, AE-BD
	2025	17	L, Q, AP, AS, BE-BS

**Table 2:** Distribution of isolates across the concentrations tested, MIC_50_, MIC_90_ and the percentage of non-wild-type (non-WT) and resistant strains for each antimicrobial. White, yellow and red boxes represent the range of concentrations tested for each antimicrobial and vertical and dotted black lines represent available ECOFFs and tentative ECOFFs, respectively. Yellow boxes indicate MIC values corresponding to the ‘intermediate’ susceptibility category, whereas red boxes represent the ‘resistant’ classification. Strains placed in gray boxes were inhibited by the lowest concentration tested (≤) or presented visible growth even at the highest concentration (>). In contrast, the symbol “-“ means that no strain was inhibited at that concentration.

Antimicrobial	MIC Values (μg/mL)																					Non-WT	Resistance
	0.015	0.03125	0.0625	0.125	0.25	0.5	1	2	4	8	16	32	64	128	250	500	256	512	1024	MIC50	MIC90		
Amoxicillin/Clavulanate *^b^				25	-	32	4	4	1	-	-									0.5	1	14%	62%
Ampicillin ^a^		-	1	1	7	8	10	3	12	8	3	13								4	>16	86%	86%
Penicillin ^c^	2	-	-	4	2	6	6	2	7	2	14	21								16	>16	97%	91%
Oxacillin ^b^				19	-	31	9	5	-	2										0.5	2	3%	3%
Cefazolin ^b^				4	-	42	15	3	1	-	1									0.5	1	3%	3%
Ceftiofur ^b^				1	-	7	40	14	2	2										1	2	6%	6%
Clindamycin ^b^					7	-	-	2	57											>2	>2	90%	90%
Enrofloxacin ^a^				61	-	-	1	-	3	1										<0.25	<0.25	8%	8%
Erythromycin ^a^		-	-	4	5	2	-	-	-	2	53									>8	>8	84%	84%
Florfenicol ^a^							19	-	32	4	11									4	>8	23%	/
Kanamycin ^b^									40	-	2	5	17	2	-	-				<8	>32	40%	36%
Rifampin ^b^		62	-	2	-	-	-	-	2											<0.0625	<0.0625	6%	3%
Tetracycline ^a^				5	-	1	-	-	-	-	2	58								>16	>16	91%	92%
Tilmicosin ^a^									12	-	2	4	48							>32	>32	82%	/
Sulfisoxazole													55	-			1	-	10	<128	<128	/	15%
trimethoprim–sulfamethoxazole **^a^			21	-	26	2	4	4	4	2	3									0.25	4	29%	14%

^a^ ECOFF defined by EUCAST for *Staphylococcus hyicus*; ^b^ ECOFF defined by EUCAST for *Staphylococcus aureus*; ^c^ ECOFF defined by EUCAST for *Staphylococcus aureus* for phenoxymethylpenicillin; * ratio 2:1; ** ratio 1:19.

**Table 3:** Annual proportions of non-wild-type (non-WT) and resistant (R) strains for tested antimicrobials interpreted with available ECOFFs and clinical BPs, respectively.

	2022		2023		2024		2025	
	Non-WT	R	Non-WT	R	Non-WT	R	Non-WT	R
Amoxicillin + Clavulanic acid	19%	56%	22%	78%	0%	58%	24%	65%
Ampicillin	81%	81%	100%	100%	79%	79%	94%	94%
Cefazolin	0%	0%	11%	11%	0%	0%	6%	6%
Ceftiofur	6%	6%	11%	11%	0%	0%	12%	12%
Clindamycin	100%	100%	100%	100%	79%	79%	88%	88%
Enrofloxacin	19%	19%	11%	11%	4%	4%	0%	0%
Erythromycin	94%	94%	89%	89%	75%	75%	82%	82%
Florfenicol	31%	NA	44%	NA	13%	NA	18%	NA
Kanamycin	44%	38%	56%	56%	38%	33%	29%	29%
Oxacillin	6%	6%	0%	0%	0%	0%	6%	6%
Penicillin	100%	88%	100%	100%	92%	83%	100%	100%
Rifampin	6%	0%	0%	0%	8%	4%	6%	6%
Sulfisoxazolo	NA	19%	NA	22%	NA	8%	NA	18%
Tetracycline	100%	100%	89%	100%	92%	92%	82%	82%
Tilmicosin	88%	NA	89%	NA	71%	NA	88%	NA
Trimethoprim/sulfamethoxazole	50%	31%	33%	11%	13%	4%	29%	12%

Note: NA: not available.

## Data Availability

The original contributions presented in this study are included in the article. Further inquiries can be directed to the corresponding author.
